# Prevalence of pharmacological adherence in patients with coronary artery disease and associated factors^*^

**DOI:** 10.1590/1518-8345.4554.3464

**Published:** 2021-09-03

**Authors:** Jaqueline Correia Padilha, Vinicius Batista Santos, Camila Takao Lopes, Juliana de Lima Lopes

**Affiliations:** 1 Universidade Federal de São Paulo, Escola Paulista de Enfermagem, São Paulo, SP, Brazil.; 2 Scholarship holder at the Coordenação de Aperfeiçoamento de Pessoal de Nível Superior (CAPES), Brazil.; 3 Universidade Federal de São Paulo, Escola Paulista de Enfermagem, Departamento de Enfermagem Clínica e Cirúrgica, São Paulo, SP, Brazil.; 4 Scholarship holder at the Conselho Nacional de Desenvolvimento Científico e Tecnológico (CNPq), Brazil.

**Keywords:** Coronary Artery Disease, Medication Adherence, Nursing, Health Education, Treatment Adherence and Compliance, Patient Compliance, Doença da Artéria Coronariana, Adesão à, Medicação, Enfermagem, Educação em Saúde, Cooperação e Adesão ao Tratamento, Cooperação do Paciente, Enfermedad de la Arteria Coronária, Cumplimiento de la Medicación, Enfermería, Educación en Salud, Cumplimiento y Adherencia al Tratamiento, Cooperación del Paciente

## Abstract

**Objective:**

to assess the prevalence of pharmacological adherence in patients with coronary artery disease and to identify factors associated with adherence.

**Method:**

a crosssectional, correlational study, including 198 patients with a previous diagnosis of coronary artery disease. Pharmacological adherence was assessed by the four-item Morisky Green test, and the factors that potentially interfere with adherence were considered independent variables. The association between the variables was determined by the Cox model, with a 5% significance level.

**Results:**

43% of the patients adhered to the treatment. Fatigue and palpitation, never having consumed alcohol and being served by medical insurance were associated with adherence. Lack of adherence was associated with considering the treatment complex, consumption of alcohol and being served by the public health care system. In the multiple analysis, the patients with fatigue and palpitations had a prevalence of adherence around three times higher and alcohol consumption was associated with a 2.88 times greater chance of non-adherence.

**Conclusion:**

more than half of the patients were classified as non-adherent. Interventions can be directed to some factors associated with lack of adherence.

## Abstract (pt)

**Objetivo:**

avaliar a prevalência da adesão farmacológica de pacientes com doença arterial coronariana e identificar os fatores associados à adesão.

**Método:**

estudo transversal, correlacional, incluindo 198 pacientes com diagnóstico prévio de doença arterial coronariana. A adesão farmacológica foi avaliada pelo teste de Morisky Green de quatro itens e os fatores que potencialmente interferem na adesão foram considerados variáveis independentes. A associação entre as variáveis foi verificada pelo modelo de Cox, com nível de significância de 5%.

**Resultados:**

43% dos pacientes aderiram ao tratamento. Associaram-se à adesão os sintomas de fadiga e palpitação, nunca ter ingerido bebida alcoólica e ser atendido por convênio médico. Associaram-se à falta de adesão considerar o tratamento complexo, o consumo de bebidas alcoólicas e ser atendido pelo sistema público de saúde. Na análise múltipla, os pacientes que apresentavam fadiga e palpitação tiveram prevalência de adesão em torno de três vezes maior e o consumo de álcool associou-se a uma chance 2,88 vezes maior de não adesão.

**Conclusão:**

mais da metade dos pacientes foram classificados como não aderentes. Intervenções podem ser direcionadas para alguns fatores associados à falta de adesão.

## Abstract (es)

**Objetivo:**

evaluar la prevalencia de la adhesión farmacológica de pacientes con enfermedad arterial coronaria e identificar los factores asociados a la adhesión.

**Método:**

estudio transversal, correlacional, que incluyó a 198 pacientes con diagnóstico previo de enfermedad arterial coronaria. Se evaluó la adhesión farmacológica mediante la prueba de Morisky Green de cuatro ítems y se consideró como variables independientes los factores que potencialmente interfieren en la adhesión. La asociación entre las variables se verificó con el modelo de regresión de Cox, con nivel de significancia del 5%.

**Resultados:**

el 43% de los pacientes adherían al tratamiento. Se asociaron a la adhesión los síntomas de fatiga y palpitaciones, nunca haber ingerido bebida alcohólica y recibir tratamiento de medicina prepaga. Se asoció a la falta de adhesión considerar complejo el tratamiento, consumir bebidas alcohólicas y recibir tratamiento del sistema público de salud. En el análisis múltiple, los pacientes que presentaban fatiga y palpitaciones tuvieron cerca de tres veces más prevalencia de adhesión y el consumo de alcohol se asoció a una posibilidad 2,88 veces mayor de no adhesión.

**Conclusión:**

se clasificó a más de la mitad de los pacientes como no adherentes. Es posible direccionar acciones hacia algunos factores asociados a la falta de adhesión.

## Introduction

According to the American Heart Association (AHA)^(1)^, 18.2 million 20-year-old or older Americans had coronary artery disease (CAD) between 2013 and 2016. In 2020, it was estimated that 720,000 Americans had some coronary event, including hospitalization for acute myocardial infarction (AMI) or death due to CAD. In Brazil, 242,858 hospitalizations occurred in 2019 due to ischemic heart disease. These data have a direct impact on economy and society^(2)^.

The treatment of CAD includes continuous use of medications, such as antihypertensives, antiplatelet agents, anticoagulants and statins. Invasive treatments can also be implemented, including coronary artery bypass grafting or percutaneous coronary intervention, in addition to the implementation of non-pharmacological measures, represented by incorporating a healthy lifestyle^(3-5)^.

Adherence to pharmacological and nonpharmacological treatment plays a crucial role in achieving satisfactory clinical outcomes in patients with CAD, including preventing ischemia, reducing symptoms, improving quality of life, decreasing readmissions and morbidity and mortality due to cardiovascular diseases^(5)^. The World Health Organization (WHO) defines adherence as the extent to which a person’s behaviour – taking medication, following a diet, and/or executing lifestyle changes, corresponds with agreed recommendations from a health care provider. There are five dimensions that interfere with this adherence: related to the patient, to the disease, the treatment, to the health care system and team, and to the socioeconomic factors^(6)^.

In developed countries, only 50% of the patients with chronic diseases continue their treatment, including pharmacological and non-pharmacological measures. In developing countries, such as Brazil, this number has varied from 51% to 56.5%^(6-8)^ when considering only pharmacological adherence. Thus, in order to contribute to the primary and secondary prevention of CAD, the multidisciplinary team must identify the factors that interfere with adherence to treatment, in order to implement interventions that may minimize these barriers^(5)^.

A Brazilian study identified that only 26% of the hospitalized patients with CAD adhere to the pharmacological treatment^(9)^. Another study found that 49.3% of the patients had low adherence to statins and antiplatelet agents or potential for such 30 days after hospital discharge for Acute Coronary Syndrome^(8)^. Lack of adherence to pharmacological treatment is a complicating factor for readmissions^(10)^ due to decompensations. A meta-analysis including 10 studies with 106,002 patients demonstrated that adequate pharmacological adherence reduced the risk of global and cardiovascular mortality, in addition to hospitalization for cardiovascular disease and AMI^(11)^.

Therefore, during the delivery of nursing care for inpatients or outpatients, nurses must assess the factors associated with pharmacological non-adherence, so that nursing interventions can be established individually and focused on these variables. To the best of the authors’ knowledge, only three Brazilian studies that evaluated the factors associated with pharmacological non-adherence in patients with CAD were identified^(7,9,12)^. Thus, new studies are needed, with population samples from different locations and addressing other possible non-adherence factors. This study aimed to assess the prevalence of pharmacological adherence in patients with CAD and to identify factors associated with adherence.

## Method

A cross-sectional, correlational study, based on the Strengthening the Reporting of Observational Studies in Epidemiology (STROBE) guidelines for crosssectional studies. It was developed in a hospital that is a reference in Cardiology in the city of São Paulo (SP), with data collection performed from November 2017 to January 2018 in the Hospitalization Units (566 beds), in the Intensive Care Units (646 beds) and in the Hemodynamics sector (51 beds).

Eligible patients were those hospitalized with a medical diagnosis of CAD, aged over 18 years. The exclusion criteria were chest pain, dyspnea or symptomatic hypotension during data collection, because these symptoms would make it impossible to interview the patients.

The sample size was calculated^(13)^ using the R 3.4.1 software, based on data from a previous study, which identified adequate pharmacological adherence in 56.5%^(7)^ of the patients with CAD. Considering an 80% power and a 10% accuracy, a minimum sample size of 198 patients was obtained.

Patients were selected by one of the authors of the study, a nurse specialized in Cardiology and Hemodynamics, who daily analyzed the patients’ medical records in the selected units and identified those who met the eligibility criteria. The nurse explained the objectives of the study and invited them to participate, by offering the free and informed consent form.

Data were collected by means of three questionnaires, in the following sequence: Morisky Green test, to assess pharmacological adherence (dependent variable); an instrument to assess the variables that interfere with patient adherence and a questionnaire to assess patient knowledge about CAD.

The four-item Morisky Green Test (Morisky Medication Adherence Scale - MMAS-4) was developed in 1986^(14)^ and is one of the most often used in Brazil^(7,15-17)^ for the indirect measurement of pharmacological adherence behavior. It consists of four questions with dichotomous answers (yes/no). The patients were considered nonadherent whenever they answered “yes” to any of the questions^(14)^. The instrument is reliable, with adequate internal consistency and stability, assessed by a Cronbach’s alpha of 0.73 (0.67-0.79) and by the test and retest (r=0.70, p=0.02), respectively^(18)^.

The independent variables were assessed through a questionnaire about the factors that can potentially interfere with adherence. The questionnaire was developed by the researchers based on the literature^(7,19)^. The factors were subdivided into five categories, according to the dimensions proposed by the WHO^(6)^:

1. patient-related variables: gender; age; ethnicity; marital status; education; number of children; religious beliefs; family assistance in the treatment (financial, company during consultation, change in eating habits and incentive to treatment – with the possibility of selecting more than one option); experience with the disease in the family context; patient’s perceived health and knowledge about the disease.
2. variables related to socioeconomic level: family income; costs of the medications purchased with own money; employment situation; type of housing and transportation costs.
3. variables related to the disease: time since diagnosis; absence of symptoms, previous hospitalization and previous invasive treatment (coronary artery bypass grafting and/or percutaneous coronary intervention).
4. treatment-related variables: undesirable effects; complex therapeutic regimens (self-declaration by the participants of considering the treatment difficult in relation to the number and frequency of medication taking); length of treatment; use of other forms of treatment and lifestyle change.
5. variables related to the health care system and team: access to the health care service; time spent to reach the health care service; waiting time for the next appointment *versus* service time and the relationship with the health care team.

Knowledge about the disease was assessed using five questions from the Questionnaire for Assessment of Knowledge in Relation to Disease, developed and submitted to content validation by Galdeano^(19)^. The questions assess familiarity with the name of the disease; description of risk factors; description of signs and symptoms; description of measures to minimize disease progression and description of signs and symptoms of disease complication^(19)^. The result was analyzed according to what was proposed by the author^(19)^: answering the entire question correctly: 1.0 point; answering half the question correctly: 0.5 points; answer a quarter of the question correctly: 0.25 points; answering the question incorrectly or not knowing how to answer it: 0 points. Thus, patients who scored ≤ 3 were considered to have deficient knowledge about the disease^(19)^.

The data were analyzed using the R 3.4.1 software and the qualitative variables were summarized by means of relative and absolute frequencies and the quantitative variables were expressed by means of measures of central tendency and dispersion [mean, standard deviation (SD), median and quartiles]. The Student’s t-test for independent samples or Mann-Whitney’s U test were used to determine the association between the quantitative variables and medication adherence, according to the distribution of the variables. Pearson’s Chi-Square test was used to determine the association of the qualitative variables with medication adherence. The Bonferroni’s correction was used to compare the alcohol intake categories two by two (never drank, stopped drinking or still drinking) and determine which comparisons were significantly different.

The Cox model with constant times and robust variance was adopted to assess the joint association between different variables and pharmacological adherence, with Prevalence Ratios (PRs) being assessed with 95% Confidence Intervals (CIs). A significance level of 5% was considered.

This study was approved by the Research Ethics Committees of the Federal University of São Paulo (Protocol No. 1.676.061) and of the *Beneficência Portuguesa* Hospital (Protocol No. 1.709.442). The study followed all legal prerogatives of research involving human beings.

## Results

A total of 198 patients were considered eligible. All agreed to participate in the study and there were no exclusions. Most were male (64.6%), married (70.2%), Catholic (62.6%), Caucasian (74.2%) and with a family history of CAD (70.7%). The mean age was 65.75 (SD=11.41 years old); the mean number of years studied was 7.27 (SD=5.41) and the mean number of children was 2.89 (SD=1.80) (Table 1).

**Table 1:** Association between pharmacological adherence and the factors related to the patient (n=198). São Paulo, SP, Brazil, 2017-2018

Variable	Pharmacological Adherence (n = 85)		Pharmacological Non-Adherence (n = 113)		p-value
	n	%	n	%	
**Gender**					
Female	31	36.5	39	34.5	0.881*
Male	54	63.5	74	65.5	
**Ethnicity**					
Caucasian	65	76.5	82	72.6	0.904*
African-American	12	14.1	20	17.7	
Asian	1	1.2	2	1.8	
Mixed (Caucasian and African-American)	7	8.2	9	8	
**Marital status**					
Not married	28	32.9	31	27.4	0.435*
Married	57	67.1	82	72.6	
**Religion**					
Catholic	51	60	73	64.6	0.221*
Evangelical	22	25.9	32	28.3	
Jehovah's Witness	3	3.5	1	0.9	
Spiritist	3	3.5	0	0	
Others	6	7.1	7	6.2	
**Family history**					
Positive	61	71.8	79	69.9	0.642*
Negative	23	27.1	30	26.5	
Does not know	1	1.2	4	3.5	
**Family assistance in treatment^‡^**					
Financial	9	11.7	11	10.6	0.815*
Companion during medical appointments consultation	68	88.3	94	90.4	0.807*
Assistance in changing eating habits	2	2.6	3	2.9	1.000*
Incentive to treatment	7	9.1	8	7.7	0.789*
**Self-perception of health**					
Excellent	1	1.2	1	0.9	
Very good	1	1.2	3	2.7	
Good	33	38.8	41	36.3	
Regular	40	47.1	56	49.6	
Poor	10	11.8	12	10.6	
**Knowledge about the disease**					
Adequate	76	89.4	99	87.6	0.824*
Deficient	9	10.6	14	12.4	
	Mean	SD	Mean	SD	
**Age (years old)**	66.61	13.09	65.1	19.97	0.378^†^
	Median	Q25;Q75	Median	Q25;Q75	
**Children**	3	2;5	3	2;4	0.499^§^
**Education (years)**	8	4;11	4	3;11	0.076^§^

SD = Standard Deviation; Q25 = Quartile 25; Q75 = Quartile 75;

* Pearson's Chi-square test;

† Student's t-test for independent samples;

‡ Variable that allows for more than one answer;

§ Mann-Whitney's U test

With regard to socioeconomic support, most patients were retired/inactive, had a family income of 1 to 3 minimum wages, had their own homes and needed family financial supplementation for the purchase of medications (Table 2).

**Table 2:** Association between pharmacological adherence and the factors related to the patient's socioeconomic support (n=198). São Paulo, SP, Brazil, 2017-2018

Variable	Pharmacological Adherence (n= 85)		Pharmacological Non-Adherence (n= 13)		p-value
	n	%	n	%	
**Family income (minimum wages)**					0.190*
Up to 1	3	3.5	6	5.3	
Between 1 and 3	34	40	60	53.1	
Between 3 and 5	15	17.6	21	18.6	
Between 5 and 7	13	15.3	9	8	
Between 7 and 9	4	4.7	6	5.3	
More than 9	16	18.8	11	9.7	
**Employment Situation**					0.449*
Retired/Inactive	54	63.5	78	69	
Active	31	36.5	35	31	
**Type of Housing**					0.254*
Own	71	83.5	87	77	
Rented	8	9.4	20	17.7	
Conceded	6	7.1	6	5.3	
**Cost of medication**					0.466*
Own money	25	29.4	33	29.2	
Social resources	13	15.3	11	9.7	
Family complementation	47	55.3	69	61.1	
**Complementation form**					0.463*
Own money	63	74.1	82	72.6	
Family complementation	9	10.6	18	15.9	
Social resources	0	0	1	0.9	
No need for complementation	13	15.3	12	10.6	
**Free transportation**					
Yes	13	15.3	24	21.2	0.358*
No	72	84.7	89	78.8	

* Pearson's Chi-Square Test

The mean time since the diagnosis was 85.1 months, ranging from 6 to 480 months. The mean duration of treatment was 82.4 months (6 - 480 months). One hundred and seventy-five patients (88.4%) had satisfactory knowledge about the disease and the mean knowledge score about the disease was 4.45 points (SD=0.83).

Most patients were non-compliant (n=113; 57.1%). Among the reasons for non-adherence, 99 (87.6%) reported forgetting to take the medication on time, 60 (53%) did not remember to take it, 26 (23%) interrupted it when they felt well and five (4.4%) reported neglecting the time to take their medications.

In the univariate analysis of the factors related to the patient and the socioeconomic situation, no significant association was found (Tables 1 and 2).

Table 3 shows that 164 patients (82.8%) had symptoms. Fatigue and palpitation were significantly associated with pharmacological adherence. With regard to invasive treatment, 158 (79.8%) underwent some type of intervention, among which 77 (48.7%) only underwent coronary artery bypass grafting, 47 (29.7%) only underwent percutaneous coronary intervention and 34 (21.5%) underwent both treatments. There was no significant association between this variable and adherence to the pharmacological treatment.

**Table 3:** Association between pharmacological adherence and the factors related to the patient's disease (n=198). São Paulo, SP, Brazil, 2017-2018

Variable	Pharmacological Adherence (n = 85)		Pharmacological Non-Adherence (n = 113)		p-value
	n	%	N	%	
**Presence of symptoms^‡^**	69	81.2	95	84.1	0.704*
Chest pain	39	56.5	61	64.2	0.335*
Dyspnea	32	46.4	42	44.2	0.874*
Fatigue	7	10.1	1	1.1	0.01*
Palpitation	6	8.7	1	1.1	0.042*
**Previous hospitalization**					
Yes	60	70.6	75	66.4	0.542*
No	25	29.4	38	33.6	
**Date of last hospitalization**					0.383*
Never been hospitalized	25	29.4	38	33.6	
Less than a year	40	47.1	57	50.4	
More than a year	20	23.5	18	16.0	
Invasive treatment^§^	66	77.6	92	81.4	0.592*
**Coronary artery bypass grafting**					0.864*
Yes	45	52.9	66	58.4	
No	40	47.1	47	41.6	
**Percutaneous coronary intervention**					1.000*
Yes	34	40.0	47	41.6	
No	51	60.0	66	58.4	
	mean	SD	Mean	%	
**Time since diagnosis (months)**	82.38	82.36	87.18	98.38	0.805^†^

SD = Standard Deviation;

* Pearson's Chi-square test;

† Student's t-test for independent samples;

‡ 164 patients reported having symptoms and could present more than one type;

§ 158 patients underwent invasive treatment. Of these, 77 underwent only coronary artery bypass grafting, 47 underwent only percutaneous coronary intervention, and 34 underwent both, totaling 111 coronary artery bypass grafting and 81 percutaneous coronary interventions

Table 4 shows that never having consumed alcohol and being served by health insurance was significantly associated with pharmacological adherence. The factors associated with lack of pharmacological adherence were the following: considering the treatment complex; having stopped drinking alcohol or still drinking alcohol and being served in the public health care service.

**Table 4:** Association between pharmacological adherence and the factors related to patient treatment (n=198), the health care service and the relationship with the health care team. São Paulo, SP, Brazil, 2017-2018

Variable	Pharmacological Adherence (n = 85)		Pharmacological Non-Adherence (n = 113)		p-value
	n	%	N	%	
**Related to the treatment**					
**Side effect**					
Yes	12	14.1	23	20.4	0.347*
No	73	85.9	90	79.6	
**Complex treatment**					
Yes	53	62.4	86	76.1	0.042*
No	32	37.6	27	23.9	
**Use of other treatments**					
Yes	3	3.5	10	8.8	0.158*
No	82	96.5	103	91.2	
**Type of complementary treatment**					0.427*
Homeopathy	1	1.16	0	0	
Home remedies	1	1.16	5	4.4	
Religion	1	1.16	2	1.8	
Others	0	0	3	2.6	
None	82	96.5	103	91.2	
**Smoking**					0.248*
Nonsmoker/passive	35	41.2	40	35.4	
Daily smoker	15	17.6	15	13.3	
Casual smoker	1	1.2	0	0	
Former smoker	34	40	58	51.3	
**Use of alcoholic beverage**					0.012*
Never drank	47	55.3	40	35.4	
Stopped drinking	30	35.3	50	44.2	
Drinks	8	9.4	23	20.4	
**Physical activity**					0.478*
Never practiced	29	34.1	46	40.7	
Practiced, but stopped	42	49.4	54	47.8	
Currently practicing	14	16.5	13	11.5	
**Related to the health care system**					
**Type of Service^§^**					
In the public system	51	60	83	73.5	0.048*
In health insurance	37	43.5	32	28.3	0.035*
Private service	18	21.2	28	24.8	0.612*
Time for access (hour)					0.712*
Up to 1	56	65.9	68	60.2	
Between 1 and 2	20	23.5	33	29.2	
More than 2	9	10.6	12	10.6	
**Date since last medical appointment (months)**					0.922*
Less than 6	76	89.4	99	87.6	
More than 6	8	9.4	12	10.6	
Does not remember	1	1.2	2	1.8	
**Relationship with the health care professional**					
Adequate	67	78.8	77	68.1	0.108*
Inadequate	18	21.2	36	31.9	
	Median	Q25;Q75	Median	Q25;Q75	
**Treatment duration (months)**	120	24;360	48	14;120	0.79^†^

Q25 = Quartile 25; Q75 = Quartile 25;

* Pearson's Chi-square test;

† Mann Whitney's U test;

§ The patients could indicate more than one type of care

In the multiple analysis, it was found that patients with fatigue and palpitation had a three-fold increase in the prevalence of medication adherence. In contrast, alcohol consumption was associated with decreased adherence, so that patients who drank had a 2.88 times greater chance of non-adherence than those who did not drink (Table 5).

**Table 5:** Multiple analysis of the factors associated with patient pharmacological adherence (n=198). São Paulo, SP, Brazil, 2017-2018

Variable	PR*	95% CI† Minimum Maximum		p-value‡
Greater than 1 to 3 minimum wages	1.168	0.374	3.649	0.790
Greater than 3 to 5 minimum wages	1.564	0.46	5.313	0.473
Greater than 5 to 7 minimum wages	2.291	0.631	8.316	0.208
Greater than 7 to 9 minimum wages	1.094	0.236	5.071	0.909
Greater than 9 minimum wages	2.493	0.608	10.22	0.204
Fatigue	3.308	1.825	5.997	0.001
Palpitation	3.294	2.177	4.983	0.001
Complex treatment	0.697	0.428	1.135	0.147
Other treatments	0.587	0.155	2.227	0.434
Stopped drinking	0.701	0.423	1.163	0.169
Drinks alcoholic beverage	0.347	0.133	0.905	0.031
Public health care system	1.194	0.613	2.322	0.603
Good relationship with the health care team	1.343	0.766	2.353	0.303
Education	1.006	0.951	1.064	0.833

* PR = Prevalence Ratio;

† CI = Confidence Interval;

‡ Cox model. Current minimum wage: R$ 937.00 (US$ 166.00)

## Discussion

This study found that most patients with CAD did not adhere to drug treatment. In addition, the majority believed that they used the prescribed medications correctly. This result can be related to the fact that the patients did not consider it lack of adherence when they forget to take their medications at the prescribed times. Other studies have also shown that a large number of patients reported neglecting the medication schedule^(8,10,20)^.

Regarding the pharmacological treatment, the mean duration of this treatment was less than the time since the diagnosis. This can occur because, at some point, many patients interrupt treatment due to lack of financial resources^(8)^, the belief that treatment would be unnecessary while they are asymptomatic or to the complex therapeutic scheme, with associated side effects^(21)^.

The proportion of patients who adhered to pharmacological treatment in this study (43%) was lower than that found in another Brazilian state, in which 56.5% of the patients with CAD were adherent to treatment^(7)^. However, patient adherence in our study was greater than that of another Brazilian study on adherence to treatment by patients with CAD (26%)^(9)^ or other chronic diseases (30.8%)^(18)^. These discrepancies reinforce the importance of further studies on the factors that can interfere with adherence to treatment by patients with CAD in Brazil, whose results may contribute to explain the differential prevalence.

Among the variables related to the disease, fatigue and palpitation were significantly associated with adherence in the univariate analysis and remained associated in the multiple analysis. Fatigue is a prevalent, disabling and persistent symptom in patients with CAD^(21)^. In a study conducted with patients undergoing percutaneous coronary intervention, this symptom was associated with the side effects of the medications^(22)^. Fatigue has also been identified as a predictor of worsening quality of life in patients newly diagnosed with CAD and in patients with chronic CAD^(4)^. This symptom also impairs psychosocial and physiological functionality^(23-24)^. Other symptoms, such as palpitation, can occur both in the initial stage of the disease, due to arrhythmias related to a recent AMI and in advanced stages of chronic CAD, due to increased areas of ischemia and consequent fibrosis^(3)^. Both symptoms generate physical discomfort, which imposes restrictions on routine habits. Thus, the individuals tend to adhere more to the treatment in order to avoid these discomforts^(25)^. On the other hand, asymptomatic patients who do not adhere to treatment report that, due to the absence of symptoms, they interrupt the medications without consulting a professional, as they feel healthy^(11)^.

Patients who had never ingested it were almost three times more likely to have drug adherence than those who had ingested it, a finding also identified previously^(8,26)^. A study^(26)^ showed that alcoholism is associated with lack of adherence to the pharmacological treatment of arterial hypertension and that patients who consume alcohol had a risk of non-adherence almost six times greater than those who do not drink. The reason would be the fear of the possible undesirable effects of the association of antihypertensive medications with alcoholic beverages.

Other variables related to adherence to treatment in the univariate analysis were the following: considering the treatment complex; assistance in the public health care system and in health care insurance. The patients who considered the treatment complex were less compliant and reported forgetting or neglecting taking medication, shortages and difficulty in their routine. The complex dosing schedule with a larger number of medications used tends to reduce adherence^(26-27)^. An important aspect of the treatment that facilitates adherence, especially in the geriatric population, is the simplification of the therapeutic regimen, with the use of drugs in fixed dose combinations in a single presentation and with a lower number of daily doses, preferably in a single dose^(28-29)^.

Regarding the access to the health care services, it was found that the patients treated by the public health care system were less compliant than those treated by health insurance companies. A study carried out with outpatients showed that those who did not have a health care plan had a 30% greater chance of not adhering to the treatment (p=0.03)^(16)^. Another study found a relationship between low adherence to treatment and assistance by the public health service (p=0.027)^(17)^.

This result can be explained by the fact that the intervals between medical appointments of public health care services are often over six months, in addition to the low professional bond caused by the turnover of professionals. A study conducted with patients with hypertension showed that the chance of low pharmacological adherence follow-up with more than one physician increased by three times^(30)^. Other studies also emphasize that the difficulty of physical access (due to distance or limited means of transportation) and the difficulties in accessing the medications also contribute to lack of adherence^(8,17)^. The individuals who have a health insurance tend to use the services more and, in turn, attend to medical appointments more often, thereby increasing the opportunity to access information that can support adherence^(10)^. One of the main benefits of easy access to the health care services is the possibility of therapeutic adjustments and monitoring^(17)^.

Multi-professional interventions and a more constant follow-up of these patients in secondary prevention programs can contribute to minimize modifiable risk factors. These factors include the use of alcohol, management of treatment complexity and inadequate understanding that the treatment is not necessary during the absence of symptoms.

Multi-professional programs have shown satisfactory results when incorporating face-to-face and telephone consultations, in addition to implementing technologies, such as sending messages to reinforce the importance of medication and/or implementing software programs with alarm sensors for medication schedules^(30-32)^. A randomized clinical trial evaluated adherence to drug treatment in three different groups (Group 1: patients in usual care; Group 2: patients who used an application software without interacting with health care professionals; Group 3: patients who used an application software and interacted with the professionals). The results showed that the patients in the groups that used the application had increased medication adherence after three months of intervention, demonstrating that technology helps the patient remember the use of the medications and is effective in increasing medication adherence^(31)^.

In the context of multi-professional programs, knowing the factors associated with lack of medication adherence helps direct educational interventions by hospital and outpatient health care professionals, with a view to adapting adherence to the pharmacological treatment of CAD and, consequently, delaying the progression of the disease, reducing new cardiovascular events and improving the patient’s quality of life.

The data in this study must be considered in the light of some limitations. First, it was carried out in a single center, which hinders the generalization of the results. In addition, the prevalence of adherence can be overestimated, since social desirability may have influenced the patients’ self-report in the Morisky Green Test. Multicenter studies in the country must be performed using objective measures of medication adherence, such as serum dosage or vials with electronic dose monitoring.

Despite its limitations, this study evaluated several variables related to adherence, as recommended by the WHO, unlike other, which assessed only a few factors that interfere with adherence. From the data identified, the need is reasserted for nurses to assess the patient globally, in the multidisciplinary context, so that they may be aware of detailed aspects related to medication adherence and establish interventions to address such factors.

## Conclusion

More than half of the hospitalized patients with CAD did not adhere to the pharmacological treatment. The factors associated with adequate pharmacological adherence were the following: fatigue and palpitation, never having consumed alcohol and being served by health care insurance. The factors associated with lack of pharmacological adherence were the following: considering the treatment complex, using or having used alcoholic beverages and being served in the public health care service. The presence of fatigue and palpitation remained as factors associated with pharmacological adherence in the multiple analysis and alcohol consumption remained as a factor associated with lack of pharmacological adherence.
